# Hotspot Identification for Shanghai Expressways Using the Quantitative Risk Assessment Method

**DOI:** 10.3390/ijerph14010020

**Published:** 2016-12-27

**Authors:** Can Chen, Tienan Li, Jian Sun, Feng Chen

**Affiliations:** 1 Department of Traffic Engineering and Key Laboratory of Road & Traffic Engineering of the Ministry of Education, Tongji University, 4800 Cao’an Road, Shanghai 201804, China; 1433975@tongji.edu.cn (C.C.); 10ironman@tongji.edu.cn (T.L.); 2 Jiangsu Province Collaborative Innovation Center of Modern Urban Traffic Technologies, SiPaiLou #2, Nanjing 210096, China

**Keywords:** expressway, hotspot identification, crash, risk assessment, potential crash costs, empirical Bayesian

## Abstract

Hotspot identification (HSID) is the first and key step of the expressway safety management process. This study presents a new HSID method using the quantitative risk assessment (QRA) technique. Crashes that are likely to happen for a specific site are treated as the risk. The aggregation of the crash occurrence probability for all exposure vehicles is estimated based on the empirical Bayesian method. As for the consequences of crashes, crashes may not only cause direct losses (e.g., occupant injuries and property damages) but also result in indirect losses. The indirect losses are expressed by the extra delays calculated using the deterministic queuing diagram method. The direct losses and indirect losses are uniformly monetized to be considered as the consequences of this risk. The potential costs of crashes, as a criterion to rank high-risk sites, can be explicitly expressed as the sum of the crash probability for all passing vehicles and the corresponding consequences of crashes. A case study on the urban expressways of Shanghai is presented. The results show that the new QRA method for HSID enables the identification of a set of high-risk sites that truly reveal the potential crash costs to society.

## 1. Introduction

Urban expressways are the backbones of the urban traffic network, which take on a large number of urban motorized traffic. According to the data collected by the Shanghai Expressway Surveillance System (SEES), from 2010 to 2013, more than 80 crashes occurred every day in the entire expressway system of Shanghai, and the average crash frequency per million vehicles per kilometer was 4.83 [1]. Congestion and travel delays are frequently caused by crashes on urban expressways in Shanghai. Thus, it is important to carry out studies on hotspot identification (HSID) to screen potentially hazardous locations on the urban expressway system.

According to the Federal Highway Administration (FHWA) report [2], 25% of the total congestion on the highway network is caused by crashes. Skabardonis et al. [3] found that freeway crashes accounted for 72% of non-recurrent congestion. That is, crashes not only result in direct losses on the relevant vehicles and occupants, but also have serious impacts on the traffic efficiency. To consider the total social losses caused by crashes in this situation, the additional travel delays caused by crashes cannot be neglected. In the United States, the extra delays caused by crashes have been considered by FHWA, which introduced the comprehensive costs of crashes [4]. The comprehensive crash costs include 11 components that referred to both the direct losses (e.g., property damage, medical cost and emergency services) and the indirect losses (e.g., travel delay and loss of quality of life). However, the comprehensive indirect losses of crashes are not obtained in China due to the information collection standard and techniques of Chinese road traffic crashes [5]. Moreover, based on the analysis of the characteristics of crashes that have occurred in Shanghai expressways, it is found that most crashes are not severe and are typically property damage only (PDO) crashes. The breakdowns caused by crashes are also commonly seen. That is, the costs of delays caused by the crashes may exceed their direct costs, which also contribute most to the total crash costs. Thus, the non-recurrent delays are adopted to express the indirect losses of crashes using the deterministic queuing diagram method.

In this paper, a new method for HSID on urban expressways is introduced based on the quantitative risk assessment (QRA) technique. The new HSID method takes account of both the direct losses and indirect losses (i.e., additional congestion delays losses) of crashes. Compared with the previous studies, the hotspots identified by this method truly reveal the potential social losses caused by crashes on urban expressways, which are more concerned with the expressway operation management department. The highlights of this study can be summarized as follows:

(1) A new HSID method is developed based on the QRA technique. The occurrence of crashes is treated as the risk, and the probability and consequences of this risk are respectively modeled. While the crash occurrence probability for all passing vehicles is expressed by the expected crash frequency at each segment, the consequences refer to the total social losses (i.e., direct losses and indirect losses) of crashes. Thus, the high-risk sites are identified, where not only many crashes occur, but also the traffic operation is heavily influenced by crashes.
(2) A classical Empirical Bayes (EB) method is used to calculate the expected crash frequency for each site. Since observed frequency and prediction of the frequency of crashes are both considered in EB, the Bayesian negative binomial model is introduced as the crash prediction model (CPM).
(3) Total social losses are used as the consequences of crashes. That is to say, the direct occupant injuries and property losses and the additional delay losses caused by a crash are both considered. The two parts of losses are quantitatively monetized. While the direct losses are estimated based on the crash type, additional delay losses are calculated using the queue theory.

The rest of the paper is organized as follows: Section 2 reviews previous studies on HSID; Section 3 provides a description of the study site and data; Section 4 introduces the QRA model; Section 5 applies a case study on the Shanghai expressway; Section 6 analyzes the results; and, finally, Section 7 concludes the paper.

## 2. Literature Review

Numerous studies on HSID have been conducted. Common HSID techniques include naive ranking according to crash frequencies or crash rates, the confidence interval technique, the crash reduction potential method, EB, and the Full Bayesian (FB) method.

Crash frequency [6] is a statistical value from historical crash data, while the crash rate [7] is obtained by normalizing the frequency of crashes with exposure (measured by traffic volume). The naive ranking methods relying on crash counts or crash rates have a significant limitation of the regression-to-mean phenomenon [8]. It means that some sites are likely to have experienced random “up” fluctuation in crashes during a short-term period of observation.

The second method for HSID is based on classical statistical confidence intervals [9]. For site i, it can be identified as unsafe if the observed crash count $K_{i}$ exceeds the observed average of counts of similar sites, u, with the level of confidence equal to δ, that is, $K_{i}>u+\delta S$, where S is the standard deviation of the group of comparison sites. The crash reduction potential method [10] uses the difference between the observed crash counts and the long-term mean of crash counts for one site as the criterion to rank high-risk sites.

To overcome the problems of naive ranking methods, Hauer et al. [11] have proposed the EB method. The EB method for HSID has been widely used as it accounts for both historical and expected crashes on the same sites—two essential elements to safety at a site. Elvik [12] has presented an extensive review of the HSID approaches and concludes that the EB method is the most reliable method. Moreover, using simulation experiments and innovative robust evaluation criteria, Cheng and Washington [13] have also proved that the EB approach is the most consistent and reliable method for HSID.

As for the FB method in road safety analysis, it has been known that FB is more capable of fitting the prior distributions [14]. Heydari et al. [15] have evaluated the effect of prior choice on the accuracy of hotspot identification and found that hotspot identification is only slightly sensitive to various prior choices. The differences between EB and FB were explored by Miranda-Moreno and Fu [16] through a simulation study in which they used the PM calculated by both methods to rank sites. They found that the FB method performed better under low-mean and small sample conditions. However, for larger data sets, the two approaches performed similarly.

Some other studies have tried to figure out the influence of crash severity on HSID. The Potential for Improvement (PFI) [17] method is another method developed for HSID. When the potential for improvement value is greater than zero, the safety of the identified location can be improved by applying appropriate measures. The univariate PFI to cases is extended when multiple response variables are modeled jointly by Sacchi et al. [18]. In addition, a new approach is proposed for multivariate FB identification and ranking of hotspots based on the Mahalanobis distance. A straightforward method is proposed in which crashes are intelligently weighted using equivalent property damage only (EPDO) crash frequency [19]. Combined with the property damage only (PDO) costs, the EPDO value summarizes the crash property costs and severity.

The index of crash cost recommended by Tarko and Kanodia [20] accounts for crash injury severity and establishes count models separately for PDOs and injuries and fatalities (I/Fs). However, it will become cumbersome to build other corresponding regression models because there are more severity classes. With considering the correlation between different crash types, Chao Wang et al. [21] have established a two-stage mixed multivariate model that combines both crash frequency and severity models to estimate the crash frequency at different severity levels. Ma et al. [22] proposed an even more complex multivariate Poisson-lognormal model to consider injury severity and frequency in a safety performance function simultaneously. However, due to its complexity, it is relatively difficult to be applied for safety managing.

In summary, previous HSID methods have selected single or multi-evaluation indexes, including crash frequency, crash injury severity, etc., to identify the potential hazardous sites. However, no previous HSID methods have considered the influence of crashes on traffic efficiency.

On the other hand, recently, quantitative risk assessment (QRA) theory has been used to evaluate the safety and efficiency of transportation systems. Meng et al. [23] proposed a QRA model to address the risk impact of traffic flow in the urban road tunnel. Tu et al. [24] have modeled travel time reliability of freeways using risk assessment techniques. In this paper, the occurrence of accidents is treated as a risk. Based on risk assessment theory, this paper proposes a safety risk assessment model that considers not only the crash frequency and injury severity, but also the non-recurrent congestion delay caused by crashes.

## 3. Study Area and Data Collection

### 3.1. Study Area

The Shanghai urban expressway system is selected as the study area. Each segment between two adjacent ramps is defined as a study unit in this paper. A total of 167 units on the system are sequentially numbered for identifying.

### 3.2. Traffic Flow Data

The traffic flow data are collected by dual loop detectors installed on Shanghai expressways. The vehicle volume, average speed and average time occupancy are acquired in a 20 s interval from the detectors.

### 3.3. Crash Data

**Figure 1:**
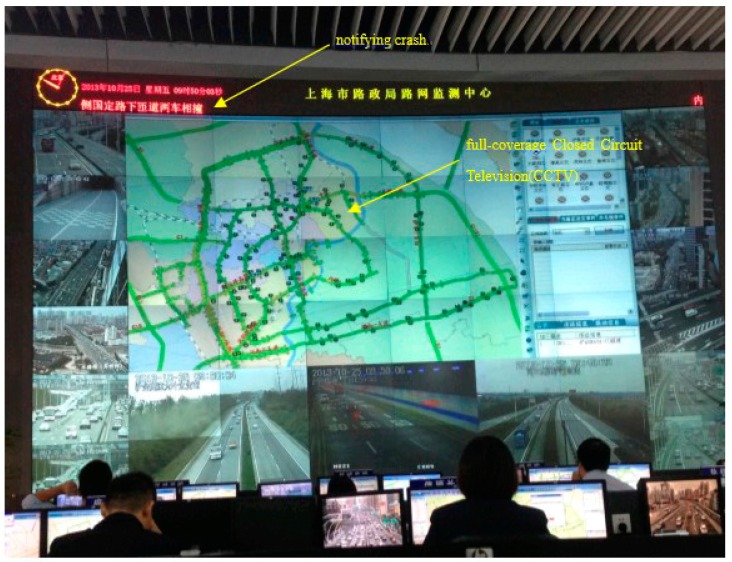
The surveillance center of Shanghai.

In total, 50,069 accident data sets were drawn from the Shanghai expressway surveillance system (SESS), as seen in Figure 1, spanning a three-year period from October 2010 to October 2013.

The crash data of SESS include basic information related to the starting time, ending time, and location of crashes. The crash locations in the SESS database are positioned to the expressway sections, which are divided by successive ramps. In addition, data for each crash include three primary crash characteristics: (1) crash type, based on the number of vehicles involved; (2) traffic state of the crash location when the crash occurred; (3) the number of lanes influenced by crashes. Due to the limitation of data collections, the crash severity (e.g., fatal, injury, and property damage only) is not recorded in the data. However, based on field investigations in the SESS, it is found that most crashes are slight, but non-recurrent congestions are frequently caused by crashes, especially in peak hours.

For the study of HSID, it is necessary to conduct an analysis of the characteristics of crashes on Shanghai expressways. The process of analysis is as follows.

**Figure 2:**
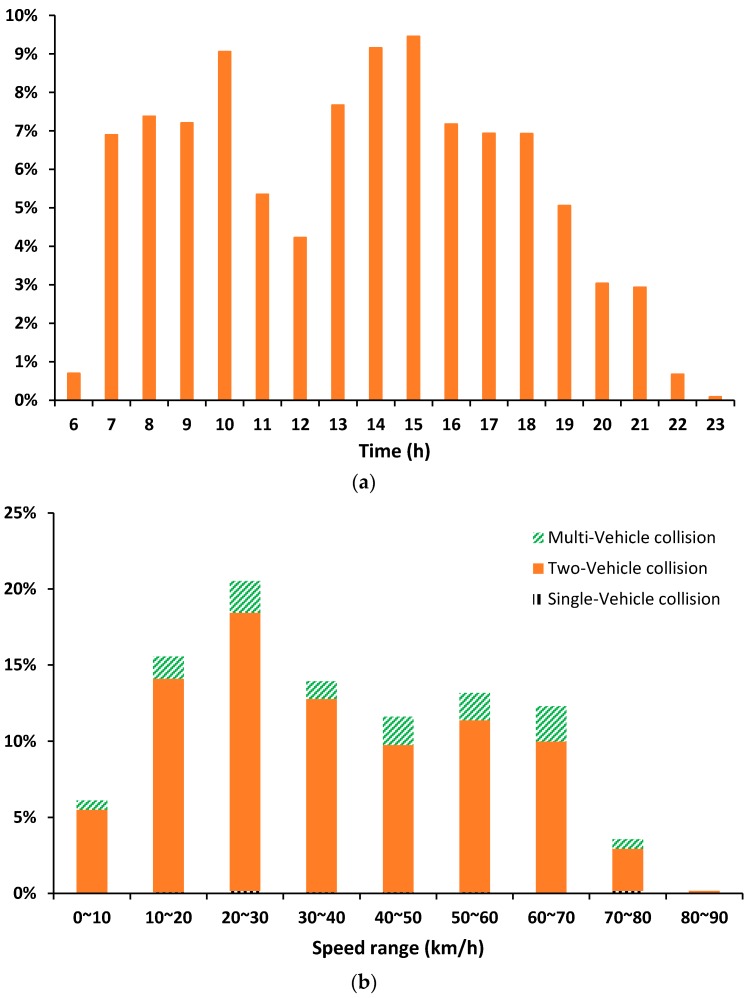
Time and speed distribution of crashes. (**a**) Time distribution of crashes; and (**b**) traffic flow speed distribution before crashes.

Figure 2a shows the time distribution of crashes. The crashes between 12:00 a.m. to 6:00 a.m. are not demonstrated because most segments on expressways are closed for maintenance during this period. Nearly 70% of crashes occurred in morning and evening peak hours of a day. Figure 2b shows the distribution of traffic flow speed five minutes before the crashes, which contain single-vehicle, two-vehicle and multi-vehicle collisions. The traffic flow speed is captured by the loop detectors at the crash location. The average speed of crashes is about 37 km/h and nearly 61% of crashes happened in crowd traffic. Thus, it is indicated that most crashes on Shanghai expressways are at low speed, which are more likely to be slight crashes.

## 4. Methodology

### Crash Risk Assessment Model

Risk assessment techniques have been widely applied in modeling accident risks, natural hazard risks, etc. [25]. The quantitative risk assessment (QRA) technique requires calculations of two components of a risk R: the potential losses L, and the corresponding probability p that the losses would occur.

The quantification of risk can be calculated through the following generalized Equation (1) (i is the index of risk, R is the measurement of risk i):

(1) $$R_{i}=L_{i}\times p(L_{i}).$$

In the case of HSID, the crashes that are likely to happen on a specific site are treated as the risk. Using the potential costs of crashes as the criteria for hotspot identification, the total risks of all vehicles passing the site are considered and the expected number of crashes for the site is used to represent the aggregation of the crash occurrence probability for all exposure vehicles. The consequences of crashes are composed of direct and indirect losses. The direct losses are the occupant injuries and property damages. The indirect losses are the additional non-recurrent congestion delays caused by crashes. The specific processes are as follows.

**Figure 3:**
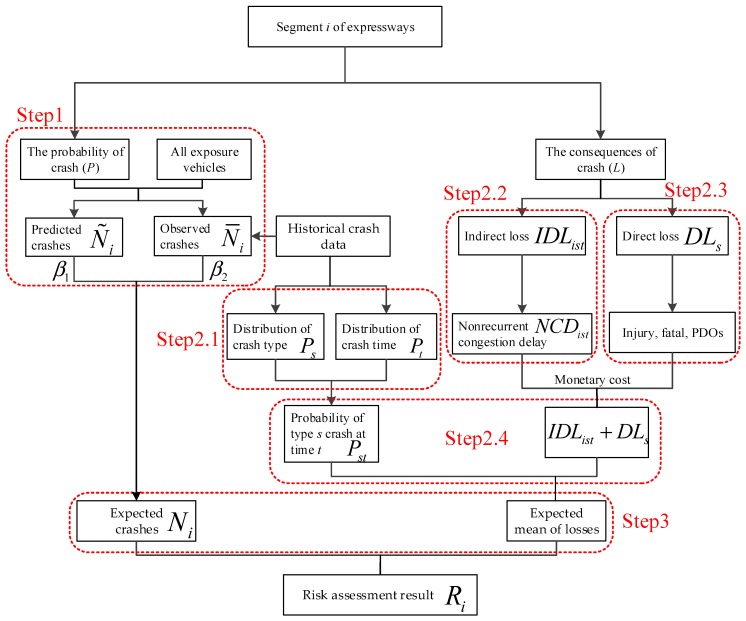
The flowchart of crash risk assessment model.

It is assumed that there are *m* segments on expressways to be assessed. The number of crash types is l. The expected crash frequency $N_{i}$ for segments i (i=1, 2···m) can be seen as the combination of crash occurrence probability p and all exposure vehicles. Crashes are assumed to be subjected to the uniform time and crash type distributions. Once the expected crash frequency $N_{i}$ is obtained, the specific occurrence time t (t=1, 2···24) and type s (s=1, 2···l) for each crash can be determined by applying the Monte Carlo sampling method [26]. Then, potential losses L related to the occurrence time t and type s can be calculated. Detailed steps are presented as shown in Figure 3 and presented as the following.

Step 1: Aggregating the Probability of Crashes for All Exposure Vehicles

The probability of crash occurrence p can be defined as the probability that a vehicle encounters with the crash when passing though the site. In addition, the aggregation of the crash occurrence probability p for all exposure vehicles can be directly expressed by the expected crash frequency with using EB method.

EB method is widely used in HSID because of its concise structure and simplicity for empirical practice. It should be noted that FB may be better especially for small samples, and it is more flexible without the limitation of prior distribution. In this paper, the crash data covers a total of 167 study sites of Shanghai expressways. Since EB can well handle such large samples [16], it is chosen to consider both observed and predicted frequencies. Whichever method is used, the time trend as indicated by historical crash counts in the study area could not be ignored, and it is assumed that there is a reduction of α% per annum. Based on EB, for a section i of expressways, the aggregation of the crash occurrence probability for all exposure vehicles can be calculated by using Equation (2):

(2) $$N_{i}=[\beta \times \tilde{N_{i}}+(1-\beta )\times \bar{N_{i}}]\times [1-n_{y}\times (\alpha /100)]$$

where $\tilde{N_{i}}$ is the predicted annual crash frequency, $\bar{N_{i}}$ is the average annual observed crash frequency at the section i over the study period, β is the weighted adjustment factor, and $n_{y}$ is the number of years in the study period.

The observed crash frequency in the analysis period can be obtained from the historical crash data. The predicted crash frequency is obtained from the CPM. CPM is used in EB to capture the relationship between the crash frequency and various explanatory variables, and provides a regression result of the crash frequency.

β is calculated as follows in Equation (3) [27]:

(3) $$\beta =1/(1+V(\tilde{N})/E(\tilde{N}))$$

where $V\left(\tilde{N}\right)$, $E\left(\tilde{N}\right)$ are the variance and expected value of the predicted crash frequency based on the study segments, respectively.

Step 2: Determining the Potential Losses of Crashes

**Step 2.1: Calculation of the probability** $P_{st}$**:** Based on the analysis of historical crash data, it is found that the proportions of the crash types do not significantly vary with time. Thus, in this study, it is assumed that the occurrence time t and type s of a crash are independent of the crash location and also independent of each other. The joint probability distribution of $P_{st}$ can be formulated as follows in Equation (4):

(4) $$P_{st}=P_{s}\times P_{t}\;(s=1,2\cdots l;\;t=1,2\cdots 24)$$

where $P_{s}$ is the probability of type *s* crash happened, and $P_{t}$ is the probability of a crash happened at time t.

While the aggregation of the crash probability has been calculated in Step 1, the Monte Carlo sampling technique is used to determine the crash type and the time for each estimated crash with the joint distribution of $P_{st}$. The Monte Carlo sampling technique uniquely defines the stochastic dynamic of the related characteristics [26]. More robust results can be obtained based on the repeated sampling.

**Step 2.2: Calculation of the indirect losses** $IDL_{ist}$**:** Several methods have been proposed for estimation of non-recurrent congestion (NCD) delays on expressways [28]. They can be classified into four groups: (1) analytical methods using deterministic queuing diagram; (2) kinematic wave; (3) heuristic method; and (4) simulation method. Compared to the other methods, the first method based on deterministic queuing diagram has been known to be concise and easy for calculation. In this paper, it is used to calculate the NCD.

**Figure 4:**
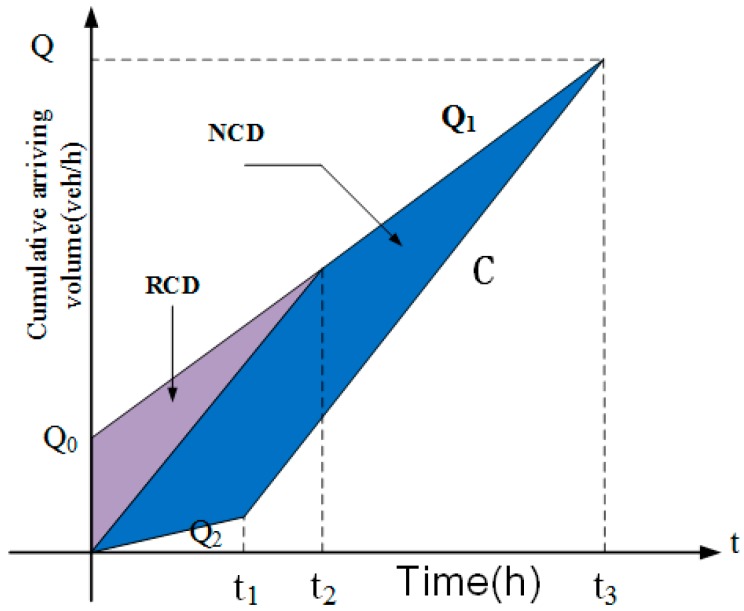
Distribution of arriving and leaving vehicles with time *t*. *NCD*: Non-Recurrent Congestion Delay; *RCD*: Recurrent Congestion Delay.

The deterministic queuing diagram method hypothesizes that arrival and discharge flow is linear to time in different phases. The distributions of arriving and leaving vehicles when a crash occurs can be demonstrated in Figure 4.

In Figure 4, NCD represents the unpredictable delay caused by the crash (h); RCD is the recurrent congestion delay (h); $t_{1}$ is the crash duration (h); $t_{2}$ is the predicted ending time of the recurrent congestion (h); $t_{3}$ is the total time of the congestion caused by the crash (h); $Q_{1}$ is the approach flow rate (veh/h); C is the maximum capacity of the specific site (veh/h); $Q_{2}$ is the operating capacity in the duration of crash (veh/h); and $Q_{0}$ is the initial queuing vehicles at the start of the crash and can be calculated by Equation (5):

(5) $$Q_{0}=\frac{L}{\bar{V}}\times {\bar{Q}}_{i}$$

where L is the total length of the segments (km), $\bar{V}$ is the average speed for the specific segment (km/h), and $\bar{Q_{i}}$ is the average volume for the specific segment (veh/h).

In general, crashes lead to the closure of some lanes and the operating capacity $Q_{2}$ will be lower than C. $Q_{2}$ can be calculated by $Q_{2}=k\times C$, where k is the reduction factor related to the number of occupied lanes [29]. The crash types used for the Shanghai expressway system are classified by involved vehicles as single-vehicle collision, two-vehicle collision and multi-vehicle collision. Generally, the more vehicles that are involved in a crash, the more lanes may be occupied. Therefore, in this study, it is assumed that the lanes occupied by crashes are related to crash types.

According to the method of deterministic queuing diagram, NCD can be calculated as follows in Equation (6) [30,31]:

(6) $$NCD=\frac{(Q_{1}-Q_{2})(C-Q_{2}){t_{1}}^{2}+2\times Q_{0}(C-Q_{2})t_{1}}{2\times (C-Q_{1})}$$

$NCD_{ist}$ can be converted into monetary costs by the average annual income c of local residents. Considering the personal average work hours $t_{h}$ per day and working days $t_{d}$ per year, the non-recurrent congestion delay $NCD_{ist}$ can be converted into costs by using Equation (7):

(7) $$IDL_{ist}=NCD_{ist}\times \frac{c}{t_{d}\times t_{h}\times 3600}$$

**Step 2.3: Calculation of the direct losses** $DL_{s}$**:** It has been mentioned that crash injury severity data is not available in the crash data, and most crashes on Shanghai expressways are slight and PDO crashes. Thus, the direct losses of a crash are assumed to be only dependent on crash type s. The value of direct losses $DL_{s}$ for various crash types s can be determined with refering to local road traffic accident statistics yearbook [32].

**Step 2.4: Calculation of the expected mean of total losses:** Based on the above steps from 2.1 to 2.3, the expected mean of total losses for section i caused by crashes in various time t and type s can be formulated as follows in Equation (8):

(8) $$\sum_{s\in l}^{l}\sum_{t=1}^{24}\left(IDL_{ist}+DL_{s}\right)\times P_{st}$$

Step 3: Calculation of the Total Risk

Based on the above results and Equation (1), the result of risk assessment for section i can be computed as follows in Equation (9):

(9) $$R_{i}=N_{i}\times \sum_{s\in l}^{l}\sum_{t=1}^{24}\left(IDL_{ist}+DL_{s}\right)\times P_{st}$$

where $P_{st}$ is the possibility of type s crashes occurring at time t. $DL_{s}$, $IDL_{ist}$ are the direct and indirect losses caused by a crash of type s occurring on section i at time t.

## 5. Case Study

Using the historical crash data and traffic flow data from expressways in Shanghai, a case study on the proposed HSID method is demonstrated.

The total number of segments m is set to be 167. The number of crash types l is set to be 3 and s=1, 2, 3 represent the single-vehicle collision, two-vehicle collision and multi-vehicle collision, respectively. As mentioned in Section 3, most segments on expressways are closed for maintenance during 12:00 a.m. to 6:00 a.m. Therefore, the occurrence time t of crashes is from 6:00 a.m. to 12:00 a.m.

Step 1: Aggregating the Probability of Crashes for All Exposure Vehicles

The observed average annual crash frequency $\bar{N_{i}}$ of segment i is obtained from the historical crash data. The predicted crash frequency $\tilde{N_{i}}$ of segment i is obtained based on the method proposed in the authors’ previous research using a Bayesian negative binomial model [1]. The historical records spanning from 2010 to 2013 suggest a nearly 8% per annum increase in crash counts, and this time trend is considered in the estimation of $\tilde{N_{i}}$ and the CPM is shown as follows:

(10) $${\tilde{N}}_{i}=LM\times ADT^{0.8410}\times e^{\left(-12.17+2.822\times CI+0.792\times MR\right)}$$

where LM is the length of the segment between two adjacent ramps (m); ADT is the average daily traffic volume of the segment (pcu/d); CI is the congestion index; and MR is the merging ratio.

The value of β is calculated to be 0.008 according to Equation (3). With the values of α and $n_{y}$, respectively, equal to 8 and 3, the expected crash frequency for each segment $N_{i}$ is then computed according to Equation (2).

Step 2: Determining the Potential Losses of Crashes

**Step 2.1: Calculation of the probability** $P_{st}$**:** To provide a robust result, the occurrence time t and type s for each expected crash are sampled by Monte Carlo sampling. The distribution of $P_{t}$ is shown in Figure 2a above. For the distribution of crash type, two-vehicle collisions occupy 87%, single-vehicle collisions occupy 12%, while multi-vehicle collisions occupy 1%. The probability distribution of $P_{st}$ is formed using Equation (4).

**Step 2.2: Calculation of indirect losses** $IDL_{ist}$**:** The value of $NCD_{ist}$ is determined by a deterministic queuing diagram method. A crash that occurred on the 52nd segment is used as an example.

**Figure 5:**
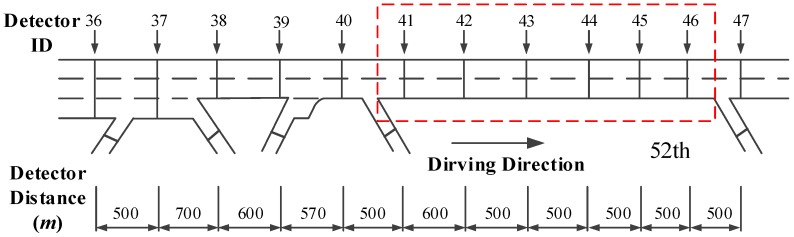
Layout of detectors on the 52nd segment.

The total length Lof the 52nd segment is 2.3 km. There are two lanes and six dual loop detectors whose ID is from 41 to 46 on the 52nd segment. The layout of detectors is shown in Figure 5. Based on the crash records, the crash started at 20:27:37 on 7 July 2010 and the crash treatment ended at 20:37:12 on 7 July 2010.

For this site, the average volume $\bar{Q}$ and speed $\bar{V}$ obtained from the central loop detectors 43 at 8:00 p.m. are, respectively, 2946 (veh/h) and 60 (km/h). The average approach flow rate $Q_{1}$ obtained from the upstream loop detectors 41 at 20 p.m. is 2988 (veh/h). The capacity C is 4078 (veh/h). The reduction factor k [29] is 0.32 and the operating capacity $Q_{2}$ during the crash is 1304 (veh/h). $t_{1}$ is calculated based on the crash record as 10 min. Using Equation (6), the NCD caused by the crash is 98.68 h.

According to the Shanghai economic operation report [33], per capita annual income c of car owners is assumed to be 80,000 yuan. Thus, the on-recurrent congestion delay $NCD_{ist}$ caused by traffic crashes can be converted to the indirect economic loss $IDL_{ist}$.

**Step 2.3: Calculation of direct losses** $DL_{s}$**:** It has been mentioned above that most crashes on expressways are PDOs. Thus, $DL_{s}$ is assumed to be only related to the number of vehicles that the crash affects. According to the Shanghai Road Traffic Accident Statistics Yearbook [32], the direct losses of a single-vehicle collision $DL_{1}$, two-vehicle collision $DL_{2}$ and multi-vehicle collision $DL_{3}$ are 2000 yuan, 4000 yuan, and 6000 yuan, respectively, in the Shanghai expressway system. As mentioned above, the direct crash losses are relatively small in the urban expressway system because most crashes are minor.

Step 3: Calculation of the Total Risk

**Figure 6:**
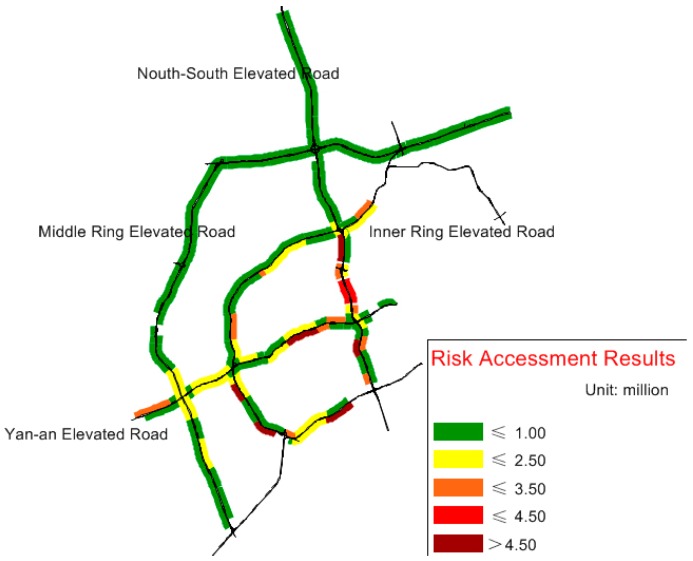
Risk assessment results of segments.

By synthesizing the other steps and Equation (9), the risk assessment results for 167 mainline segments of Shanghai Expressway are shown in Figure 6.

## 6. Results and Analysis

**Figure 7:**
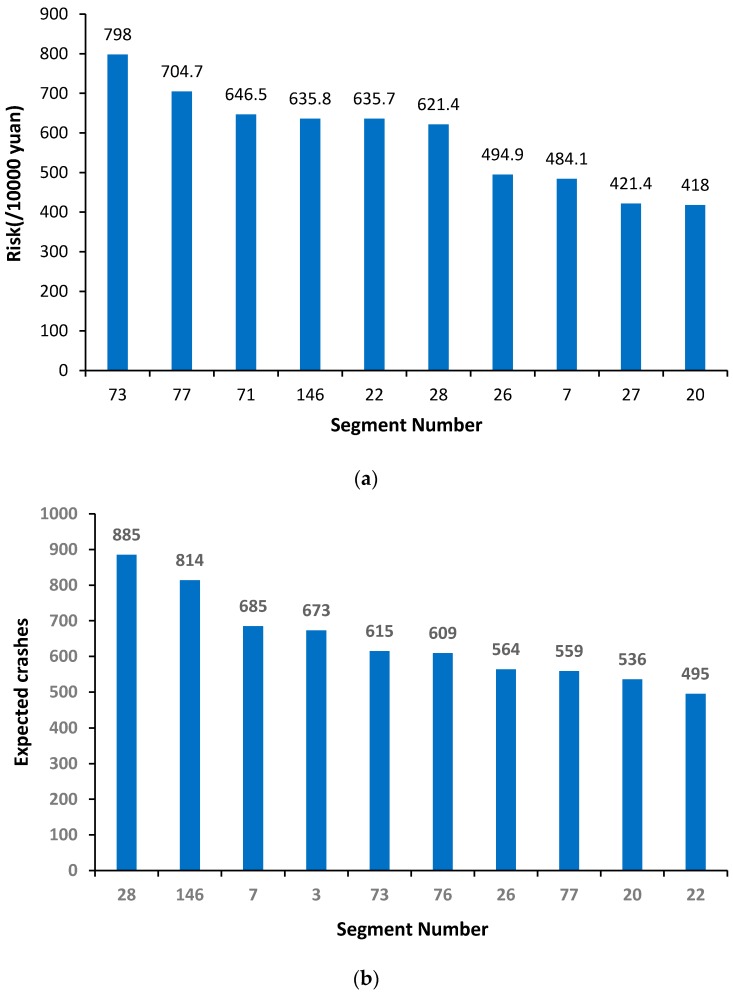
Top 10 hotspots based on quantitative risk assessment (QRA) and empirical Bayesian (EB). (**a**) top ten hotspots based on risk assessment; and (**b**) top ten hotspots based on the EB method.

The top ten sites determined by the new QRA method and conventional EB method are, respectively, shown as follows Figure 7a,b.

Figure 7 shows that the rankings of the top 10 hotspots identified by the two methods have a significant difference. For example, the 28th segment is the first one in EB, while it turns out to be the sixth one in QRA. Meanwhile, the order of the 73rd segment rises from the fifth in EB to the top one in QRA. These two segments are used as an example to conduct further discussion.

**Figure 8:**
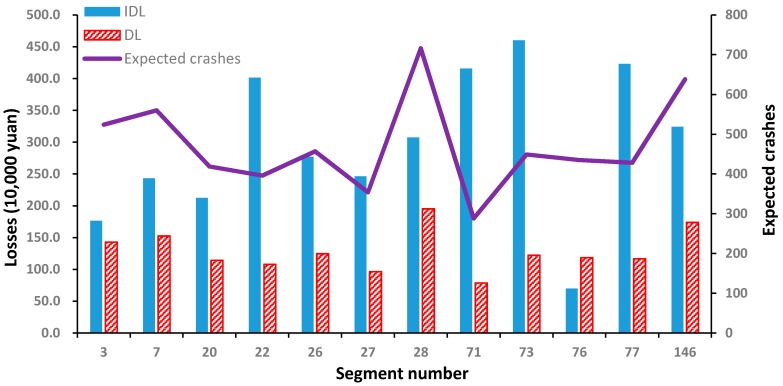
Relationships between $N_{i}$ and $DL_{i}$/$IDL_{i}$ for partial segments.

As shown in Figure 8, the expected crashes of the 28th segment are 270 higher than that of the 73rd segment. Thus, the direct losses are 0.74 million yuan more than that of the 73rd segment. The average direct losses are only about 2.7 thousand yuan for each crash. On the contrary, the indirect losses of the 73rd segment are about 2.5 million yuan higher than that of the 28th segment, and the average indirect losses are nearly 2.4 times as much as that of the 28th segment.

Through local observation, it is found that there are only two lanes for the 73rd segment while the traffic demand there is large. Once a crash occurs, the capacity drops significantly and massive additional non-recurrent congestion delays may occur on the 73rd segment. As expected, the new QRA method places more emphasis on indirect losses caused by crashes, and the 73rd segment is identified to be the top hotspot.

**Figure 9:**
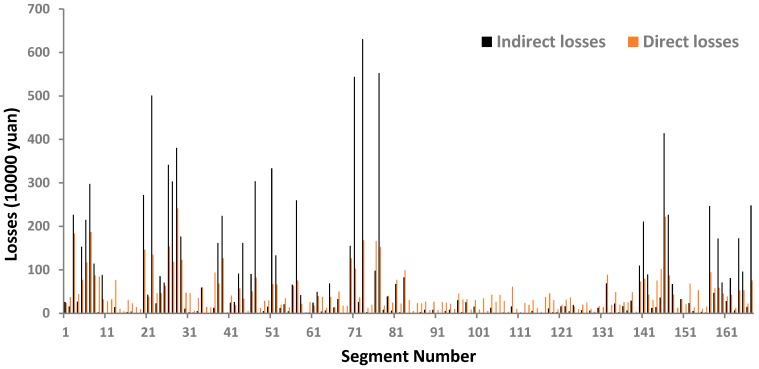
Indirect and direct losses of different segments.

Figure 9 shows the indirect and direct losses of all of the studied segments. The indirect losses of most segments are higher than the corresponding direct losses. That is, without considering the indirect losses, the results of conventional methods, such as EB, are likely to be biased. Thus, in order to improve both safety and operational efficiency of expressways, it is necessary to consider the indirect losses caused by crashes for HSID.

## 7. Conclusions 

The occurrence of traffic crashes is a severe hazardous situation on the urban expressway operation. It not only results in direct losses on the vehicles and occupants, but also seriously influences the efficiency of the expressway. To screen potentially hazardous locations considering the total social losses of crashes, a QRA method for HSID on urban expressways is developed based on the crash and traffic flow data in a three-year period in Shanghai. The main conclusions are as followings:

(1) The use of the QRA method enables the identification of a set of high-risk sites that reveal the potential total crash costs to society. The case study results show that the rankings of hotspots are different between the conventional EB method and the new QRA method.
(2) In the QRA framework for the probability of crashes, in order to take full account of the uncertainty existing between roadway characteristics and crashes, EB combined with the Bayesian negative binomial model is used to calculate the expected number of crashes. It is shown that the classical EB is applicable and provides a robust result for the probability of crashes.
(3) In the QRA framework, for the consequences of crashes, the equivalent monetary index is applied to unify the direct and indirect losses. Indirect losses of crashes are quantitatively estimated by using the queue theory. The traffic situation when crashes happen and the crash type are sampled using Monte Carlo sampling.

The QRA method proposed in this paper is suggested especially for the situations in which the congestions caused by crashes should not be ignored. The potential crash costs to society can thus be truly revealed for the department of safety management. Future works are suggested to further study the reliability of the method. The method can also be expanded by applying some more advanced techniques like the FB method [14], and considering spatial effects [34,35] and other potential patterns.
